# Late Postpartum Eclampsia with Posterior Reversible Encephalopathy Syndrome and Subarachnoid Hemorrhage: A Case Study

**DOI:** 10.3390/medicina61010077

**Published:** 2025-01-05

**Authors:** Mohamed Elshafei, Hala Ashraf Oweis, Yosra Abdul Hafez, Thuraya Alom, Zahraa Mohamed Hayani, Eslam ElNebrisi

**Affiliations:** 1 Department of Neurology, Zulekha Hospital, Dubai 48577, United Arab Emirates; dr.link147@yahoo.com; 2 Department of Internal Medicine, Dubai Medical College for Girls, Dubai 20170, United Arab Emirates; haa20200125@dmcg.edu (H.A.O.); yhs20200167@dmcg.edu (Y.A.H.); tha20200164@dmcg.edu (T.A.); zmh20200169@dmcg.edu (Z.M.H.); 3 Department of Biomedical Sciences, Dubai Medical College for Girls, Dubai 20170, United Arab Emirates

**Keywords:** posterior reversible encephalopathy syndrome, subarachnoid hemorrhage, eclampsia

## Abstract

Eclampsia is a multisystem disorder of pregnancy and the puerperium. Posterior reversible encephalopathy syndrome (PRES), a neurotoxic condition characterized by various neurological symptoms, can arise from multiple causes including eclampsia. Although hemorrhage is a possible complication of PRES, subarachnoid hemorrhage (SAH) is a rare occurrence in eclamptic patients with this condition. A 33-year-old female patient presented with acute severe headache and blurred vision two days after delivery. This progressively worsened over the next five days before she was admitted to the hospital. A magnetic resonance imaging (MRI) brain scan with contrast revealed a picture suggestive of PRES. Following admission, she had seizures, and a follow-up MRI revealed an acute subarachnoid hemorrhage. Treatment started, and the patient improved and was discharged from the hospital without any residual symptoms. This case illustrates how eclampsia can be a risk factor for PRES, and although hemorrhagic PRES is becoming more recognized, SAH remains an unusual but critical presentation. Early and accurate diagnosis, along with effective management, is crucial for achieving a positive outcome.

## 1. Introduction

Eclampsia is a severe, potentially life-threatening complication often associated with hypertensive disorders during pregnancy. It is characterized by endothelial dysfunction, systemic inflammation, and cerebral autoregulation disruption, which collectively contribute to increased vascular permeability and vasogenic edema [1,2]. These processes lead to neurological symptoms such as seizures and visual impairments, including cortical blindness, blurred vision, and scotomas. While eclampsia commonly occurs from the 20th week of gestation to 48 h postpartum, cases of late-onset eclampsia—occurring up to 4 weeks after childbirth—have also been documented [3]. Rare instances, such as eclampsia manifesting up to 6 weeks postpartum, have been reported in the literature, further emphasizing its unpredictability [4].

Posterior reversible encephalopathy syndrome (PRES) is an acute neurotoxic condition marked by various neurological and radiological features, often triggered by factors such as hypertension, sepsis, eclampsia, autoimmune diseases, and immunosuppressive treatments [1,5,6]. The characteristic subcortical white matter edema seen in PRES results from the disruption of the blood–brain barrier (BBB), primarily caused by endothelial dysfunction. Severe hypertension or other triggers of PRES impair the autoregulatory mechanisms of the cerebral vasculature, leading to vasodilation and hyperperfusion. This hyperperfusion increases hydrostatic pressure, which compromises the integrity of the BBB and allows protein-rich fluid to leak into the interstitial space, resulting in vasogenic edema predominantly in subcortical white matter [5,7]. Additional contributors include postpartum hemorrhage, hormonal fluctuations, and massive blood transfusions [1,8]. Clinically, PRES presents with headaches, visual impairments, altered consciousness, and seizures. Imaging typically reveals subcortical white matter edema predominantly in the parieto-occipital region [5,9].

While intracranial hemorrhage is a recognized complication of PRES, subarachnoid hemorrhage (SAH) is rare. Typically, hemorrhages in PRES are small or moderate and spare the basal cisterns, distinguishing them from aneurysmal SAH. Hemorrhagic complications in PRES, particularly SAH, are associated with increased morbidity and mortality, highlighting the importance of early diagnosis and treatment [10,11,12]. This report describes a case of late postpartum eclampsia complicated by PRES and SAH, detailing the diagnostic approach and management strategies.

## 2. Case Presentation

Our patient, a 33-year-old Asian woman (Gravida 1, Para 1), presented with severe holocephalic headache, blurred vision, and nausea two days after delivering her first baby via cesarean section (CS) under spinal anesthesia. Her symptoms were not accompanied by vomiting, photophobia, phonophobia, or diplopia. The headache, aggravated by physical activity and lying supine, disrupted her sleep despite oral paracetamol treatment. The patient was referred to the neurology department after her symptoms worsened.

Her past medical history included infrequent migraines and familial hypercholesterolemia, with no prior history of similar headaches, fever, rash, joint pain, or neurological disorders. Antenatal screenings revealed only microcytic hypochromic anemia, with no gestational hypertension, diabetes, or infections. Delivery was uncomplicated, and postoperative recovery was uneventful.

On admission, her vital signs included BP = 145/95 mmHg (elevated); HR = 95 bpm; T = 36.2 °C; RR = 20 breaths/min; and BMI = 34.3 kg/m^2^ (obese). General examination showed mild bilateral ankle edema, but the cesarean scar was clean. Neurological examination revealed a conscious, oriented patient with normal cranial nerve function, visual acuity, motor strength, and reflexes. However, the patient exhibited severe distress due to her headache.

**Figure 1:**
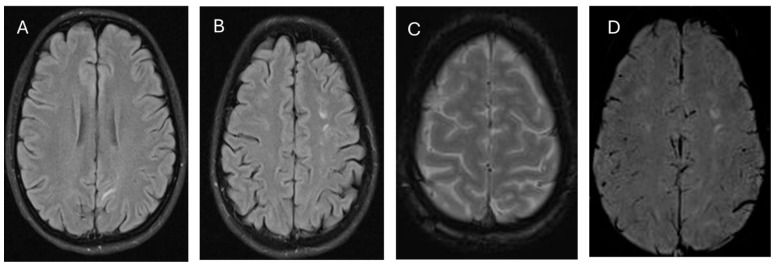
MRI brain (**A**,**B**): FLAIR showed multiple focal T2W/FLAIR hyperintensities in the subcortical white matter of the frontoparietal and occipital lobe. (**C**): MAG showed no apparent hemorrhage. (**D**): SWI showed multiple focal T2W/FLAIR hyperintensities in the subcortical white matter of the frontoparietal and occipital lobes.

Initial imaging with brain MRI revealed bilateral parieto-occipital hyperintensities consistent with vasogenic edema (Figure 1A,B). MR angiography excluded venous sinus thrombosis and vascular spasm (Figure 1C,D). Overnight, her blood pressure fluctuated between 140 and 180 systolic and 90 and 110 diastolic. Despite starting amlodipine, her headache persisted. On the second day of admission, she experienced a generalized tonic–clonic seizure lasting three minutes, which resolved with diazepam. She was subsequently started on intravenous magnesium sulfate.

**Figure 2:**
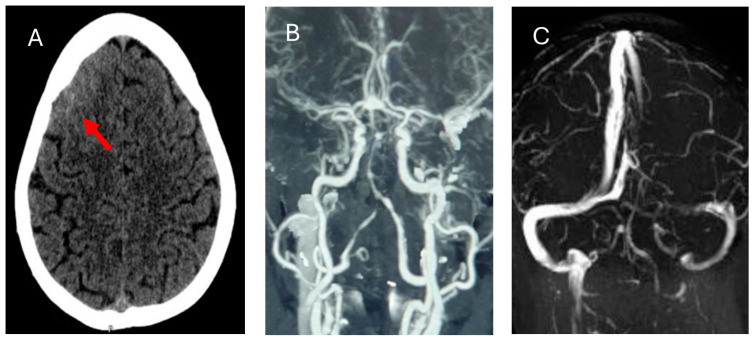
CT brain (**A**): CT BRAIN showed subarachnoid hemorrhage in the right frontal lobe (red arrow) and multiple small petechial parenchymal hemorrhages in the right frontal lobe. (**B**,**C**): CT angiogram (arterial and venous) showed no evidence of intracranial aneurysm or vascular malformation and no evidence of cerebral venous sinus thrombosis with hypoplastic left transverse sinus.

**Figure 3:**
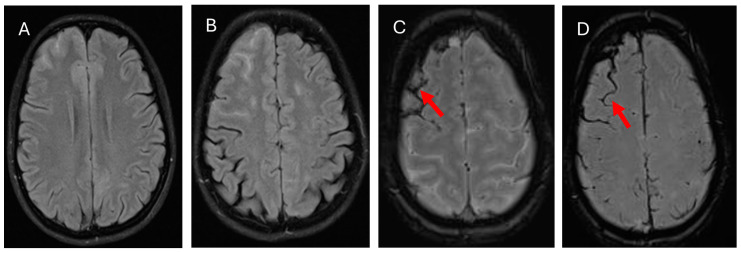
MRI brain (**A**,**B**): FLAIR showed multiple focal T2W/FLAIR hyperintensities in the subcortical white matter of the frontoparietal and occipital lobes and evidence of recent right frontal subarachnoid and petechial hemorrhage. (**C**): MAG showed right frontal subarachnoid hemorrhage (red arrow). (**D**): SWI showed multiple focal T2W/FLAIR hyperintensities in the subcortical white matter of the frontoparietal and occipital lobes and recent right frontal subarachnoid and petechial hemorrhage.

Follow-up CT imaging after the seizure revealed a right frontal subarachnoid hemorrhage (Figure 2A). CT angiography showed no evidence of aneurysm, vascular malformation, or cerebral venous sinus thrombosis (Figure 2B,C). MRI confirmed the presence of vasogenic edema and a right frontal SAH (Figure 3A–D). Lumbar puncture findings were notable for mild protein elevation (CSF protein: 65 mg/dL) with normal glucose levels and an opening pressure of 12 cm H_2_O (Table 1). CSF analysis and viral PCR testing excluded infectious causes. Laboratory workup revealed proteinuria and hyperlipidemia but no evidence of coagulopathy or significant metabolic abnormalities (Table 1).

The patient responded well to treatment and experienced no further seizures. She was discharged with levetiracetam and antihypertensives, and follow-up imaging after one month confirmed resolution of the vasogenic edema and SAH, with residual hemosiderin deposition.

This case report adheres to the CARE (CAse REport) guidelines to ensure clarity and completeness in reporting (Table S1).

## 3. Discussion

This case represents a unique and complex instance of late postpartum eclampsia complicated by posterior reversible encephalopathy syndrome (PRES) and subarachnoid hemorrhage (SAH). The rarity of this presentation adds valuable insights to the current body of literature and underscores the importance of prompt diagnosis and interdisciplinary management. Late-onset eclampsia, occurring more than 48 h postpartum, is a rare but significant clinical entity [13,14]. Unlike typical cases that manifest within the immediate peripartum period, delayed presentations can pose diagnostic challenges, particularly in patients without traditional risk factors such as pre-eclampsia or severe hypertension [1,2,12]. This case highlights the need for vigilance in postpartum patients presenting with new-onset neurological symptoms, even in the absence of classical predisposing factors. PRES, first described by Hinchey et al., is characterized by vasogenic edema due to blood–brain barrier (BBB) disruption and endothelial dysfunction [1,8,15]. The classic imaging pattern of PRES involves symmetrical hyperintensities in the parieto-occipital region, attributed to the vulnerability of the posterior circulation to fluctuations in blood pressure [16,17]. However, atypical imaging patterns, including frontal or cerebellar involvement as seen in this case, highlight the diagnostic variability and complexity of the syndrome [18]. Understanding these less common presentations is crucial for timely and accurate diagnosis.

The mechanisms underlying PRES and SAH are complex and multifactorial. Two prominent theories provide insight into the development of SAH in PRES: (1) Hypertension-Induced Vessel Rupture: Severe fluctuations in blood pressure can lead to shear stress on pial vessels, predisposing them to rupture and resulting in SAH. In this case, intermittent hypertensive surges during hospitalization likely contributed to vascular compromise [19]. (2) Ischemia–Reperfusion Injury: PRES-related endothelial dysfunction disrupts the blood–brain barrier (BBB), leading to vasogenic edema. Ischemia and subsequent reperfusion further weaken vessel integrity, promoting hemorrhage. While hemorrhages in PRES are typically petechial or intraparenchymal, the occurrence of SAH underscores the dynamic nature of vascular injury in this syndrome [20,21]. These mechanisms highlight the complex interplay between systemic and local vascular factors, underlining the importance of continuous monitoring and individualized management in such cases. Further research is needed to elucidate the precise molecular pathways contributing to these complications, which may pave the way for targeted interventions.

This case demonstrated atypical imaging findings, including frontal involvement in PRES, alongside a rare occurrence of localized SAH. Compared to similar cases, the unique combination of clinical and radiological features in this patient enriches the understanding of the variability of PRES. Table 2 summarizes key findings from this and comparable cases, providing a comprehensive view of clinical presentations and outcomes.

A systematic approach to differential diagnosis was essential in this case to rule out other conditions that may mimic PRES-associated SAH. Common considerations included cerebral venous sinus thrombosis (CVST), aneurysmal SAH, hypertensive encephalopathy, and infectious meningoencephalitis. MR angiography excluded venous sinus thrombosis, and imaging findings did not support aneurysmal rupture. Infectious causes were excluded through negative viral PCR and normal CSF glucose levels, while the distinct imaging patterns confirmed PRES over hypertensive encephalopathy.

From a clinical perspective, this case underscores several key lessons:

Late-Onset Eclampsia and PRES: Clinicians must maintain a high index of suspicion for eclampsia and PRES, even in the late postpartum period, as early recognition is critical for improving outcomes.

Dynamic Nature of PRES: The evolving nature of PRES, with delayed complications such as SAH, necessitates regular imaging follow-up during treatment to monitor for new developments.

Tailored Management: Individualized treatment strategies should consider the patient’s unique risk factors and clinical presentation, emphasizing the importance of personalized care in complex cases.

This case adds to the limited literature on PRES-associated SAH in eclampsia, highlighting the need for further research. Understanding the mechanisms underlying these complications and identifying predictive factors can improve diagnosis and management in similar cases.

## 4. Conclusions

This case highlights the rare and complex presentation of late-onset eclampsia complicated by PRES and SAH. While eclampsia is typically expected within the immediate postpartum period, its delayed manifestation poses significant diagnostic challenges. The dynamic nature of PRES, with delayed complications such as SAH, underscores the importance of continuous monitoring and timely intervention. Early recognition and individualized management are critical to achieving favorable outcomes. This case adds to the growing body of evidence emphasizing the need for vigilance in postpartum care and the reversibility of PRES with prompt treatment.

Clinical Implications:

- Healthcare providers must maintain a high index of suspicion for eclampsia and its complications, even in the late postpartum period, especially in patients presenting with neurological symptoms.
- The evolving nature of PRES necessitates regular imaging follow-up during treatment to identify delayed complications such as SAH.
- Individualized treatment plans that address patient-specific risk factors, including vascular dysfunction, are essential for managing rare presentations like PRES-associated SAH.
- Effective management requires collaboration across neurology, obstetrics, and radiology to ensure comprehensive care and optimize outcomes.
- Further studies are essential to better understand the pathophysiology of PRES-associated SAH, identify predictive factors, and develop targeted interventions for similar cases.

## Figures and Tables

**Table 1:** Laboratory findings in our case study.

CBC	N	Protein/creatinine ratio	0.5 (H)	INR	1.05 (N)	ANA	-ve
Liver	N	24 h urinary protein	3.5 gm (H)	APTT	32 (N)	Anti-ribonucleoprotein antibody	-ve
Renal	N	Urine analysis and culture					
Thyroid	N	Wound swab and culture	-ve	D-Dimer	192 (H)	RF	-ve
HBA1C	N	COVID-19	-ve	TG	276 (H)	Antithyroglobulin antibody	-ve
Uric acid	6.5 (N)	HSV	-ve	Cholesterol	447 (H)	Anti-thyroid peroxidase antibody	-ve
ESR	24 (N)	HZV	-ve	LDL	342 (H)	Anti-Smith antibody	-ve
CRP	21 (H)	CMV	-ve	HDL	60 (N)	Anti-cardiolipin antibody	-ve
CSF-Protein	65 (H)	HTLV-1	-ve	CSF-opening pressure	12 Cm H_2_O	Myeloperoxidase anti-neutrophil cytoplasmic antibody	-ve
CSF-Cell	1 (lymphocyte)	HIV	-ve	IgG4	-ve	Proteinase 3 anti-neutrophil cytoplasmic antibody	-ve
CSF-Glucose	60 (N)	EPV	-ve	Sjogren’s syndrome antibody type A	-ve	Sjogren’s syndrome antibody type B	-ve

Table abbreviations: N: normal; -ve: negative; H: high.

**Table 2:** Comparative analysis of PRES with SAH cases.

Study	Clinical Features	Imaging Findings	Management	Outcome
Hu et al. (2018) [11]	Severe headache, seizures	Parieto-occipital edema, focal SAH	Antihypertensives	Complete resolution
Masai et al. (2019) [2]	Altered consciousness, blurred vision	Bilateral parieto-occipital hyperintensities	Anticonvulsants, BP control	Residual neurological deficits
Bender et al. (2024) [22]	Severe headache, altered consciousness	Extensive vasogenic edema, intraparenchymal hemorrhage	Supportive care, BP control	Partial recovery
Present Case	Severe headache, blurred vision, seizures	Parieto-occipital hyperintensities, focal SAH	Magnesium sulfate, levetiracetam, antihypertensives	Complete resolution

## Data Availability

The original contributions presented in the study are included in the article/Supplementary Materials; further inquiries can be directed to the corresponding author.

## Supplementary Materials

The following supporting information can be accessed and downloaded at https://www.mdpi.com/article/10.3390/medicina61010077/s1: Table S1: CARE checklist—items to include when reporting case studies.
