# Supplementary material for: Late Postpartum Eclampsia with Posterior Reversible Encephalopathy Syndrome and Subarachnoid Hemorrhage: A Case Study
Source: Medicina (Kaunas). 2025 Jan 5;61(1):77. doi: 10.3390/medicina61010077 (PMC11767126; doi:10.3390/medicina61010077)
Supplement: Supplementary file 1 [file medicina-61-00077-s001.zip › medicina-3383745-supplementary.pdf]

CARE guidelines for case reports: 13-item checklist

Please indicate in which section each item has been reported in your manuscript. If you feel that an item does not apply to your manuscript, please enter N/A.

For more information about the CARE guidelines, please see http://www.care-statement.org/.

No.	Description	Section #
**Title**		
1	The area of focus and "case report" should appear in the title	line 3
**Keywords**		
2	Two to five key words that identify topics covered in this case report	25
**Abstract**		
3a	Introduction—What is unique about this case? What does it add to the medical literature?	150-163
3b	The main symptoms of the patient and the important clinical findings	50-66
3c	The main diagnoses, therapeutics interventions, and outcomes	67-105
3d	Conclusion—What are the main 'take-away' lessons from this case?	165-186
**Introduction**		
4	Briefly summarize why this case is unique with medical literature references	27-48
**Patient information**		
5a	De-identified demographic information and other patient specific information	50-51
5b	Main concerns and symptoms of the patient	50-55
5c	Medical, family, and psychosocial history including relevant genetic information	56-58
5d	Relevant past interventions and their outcomes	59-60
**Clinical findings**		
6	Describe the relevant physical examination (PE) and other clinical findings	61-66
**Timeline**		
7	A timeline of relevant information from the patient's history and this episode of care	NA
**Diagnostics assessment**		
8a	Diagnostic methods (such as PE, laboratory testing, imaging, surveys)	67-81
8b	Diagnostic challenges (such as access, financial, or cultural)	NA
8c	Diagnostic reasoning including a differential diagnosis	111-127
8d	Prognostic characteristics (such as staging in oncology) where applicable	129-134
**Therapeutic intervention**		
9a	Types of intervention (such as pharmacologic, surgical, preventive, self-care)	137-142
9b	Administration of intervention (such as dosage, strength, duration)	72-73
9c	Changes in intervention with rationale	137-139
**Follow-up and outcomes**		
10a	Clinician and patient-assessed outcomes when appropriate	102-104
10b	Important follow-up diagnostic and other test results	79-86
10c	Intervention adherence and tolerability (how was this assessed?)	102-104
10d	Adverse and unanticipated events	NA
**Discussion**		
11a	Discussion of the strengths and limitations in your approach to this case	106-111
11b	Discussion of the relevant medical literature	114-149
11c	The rationale for conclusions (including assessment of possible causes)	126-136
11d	The primary "take-away" lessons of this case report	150-163
**Patient perspective**		

12	When appropriate, the patient can share their perspective on their case	NA
**Informed consent**		
13	The patient should give informed consent	198

When submitting your manuscript via the online submission form, please upload the completed checklist as a Figure/supplementary file.

If you would like this checklist to be included alongside your article, we ask that you upload the completed checklist to an online repository and include the guideline type, name of the repository, DOI and license in the *Data availability* section of your manuscript.

Developed from: Riley DS, Barber MS, Kienle GS, AronsonJK, von Schoen-Angerer T, Tugwell P, Kiene H, Helfand M, Altman DG, Sox H, Werthmann PG, Moher D, Rison RA, Shamseer L, Koch CA, Sun GH, Hanaway P, Sudak NL, Kaszkin-Bettag M, Carpenter JE, Gagnier JJ. CARE guidelines for case reports: explanation and elaboration document. J Clin Epidemiol. 2017 May 18. pii: S0895-4356(17)30037-9. https://doi.org/10.1016/j.jclinepi.2017.04.026
